# Molecular mechanisms involved in *Plasmodium* gametocytogenesis

**DOI:** 10.3389/fmicb.2026.1736981

**Published:** 2026-02-04

**Authors:** Aline Miranda Scovino, Rafaella Stéfany Oliveira-da-Silva, Joyce Almeida-da-Silva, Karolynne Dantas Mendes, Elias Barbosa da Silva-Junior, Debora Decote-Ricardo, Leonardo Freire-de-Lima, Celio Geraldo Freire-de-Lima, Paulo Renato Rivas Totino, Alexandre Morrot

**Affiliations:** 1 Immunoparasitology Laboratory, Oswaldo Cruz Institute (FIOCRUZ), Rio de Janeiro, Brazil; 2 Faculty of Medicine, Federal University of Rio de Janeiro (UFRJ), Rio de Janeiro, Brazil; 3 Carlos Chagas Filho Institute of Biophysics, Federal University of Rio de Janeiro (UFRJ), Rio de Janeiro, Brazil; 4 Veterinary Institute of the Federal Rural University of Rio de Janeiro (UFRRJ), Seropedica, Brazil; 5 Laboratory for Malaria Research, Oswaldo Cruz Institute (FIOCRUZ), Rio de Janeiro, Brazil

**Keywords:** epigenetic regulation, gametocytogenesis, malaria, *Plasmodium*, post-transcriptional regulation, transcription regulation

## Abstract

Malaria, a disease caused by protozoa of the genus *Plasmodium*, remains a major challenge for global public health. The persistence of disease transmission to the mosquito vector depends on the differentiation of asexual blood-stage parasites into gametocytes, a process known as gametocytogenesis. Interrupting this stage of the parasite’s life cycle represents a critical strategy for malaria control and eventual eradication. This review aims to consolidate recent advances in the understanding of the complex molecular mechanisms regulating gametocytogenesis in *Plasmodium*, with a particular focus on *P. falciparum*. Sexual differentiation is modulated by various factors, including environmental stressors such as the depletion of lysophosphatidylcholine (LysoPC), and is orchestrated through a sophisticated regulatory network. At the transcriptional level, the AP2-G transcription factor functions as a master switch, whose expression is tightly regulated by epigenetic mechanisms, including histone H3K9 trimethylation (H3K9me3) as well as the activity of both heterochromatin protein 1 (HP1) and gametocyte development protein 1 (GDV1). Following commitment, post-transcriptional regulation plays a critical role in further differentiation, including transcript stabilization by RNA-binding proteins such as PfPuf1 and PfPuf2, along with epitranscriptomic modifications such as mRNA methylation (m^5^C and m^6^A), which modulate gene expression. A comprehensive understanding of these interconnected regulatory pathways is essential for the identification of novel therapeutic targets and the development of effective transmission-blocking vaccines.

## Introduction

According to the World Health Organization (WHO), in 2023 there were 263 million cases of malaria in the world, an increase of 11 million cases compared with 2022. Between 2019 and 2021, an additional 13.4 million cases were attributed to disruptions in anti-malaria actions during the COVID-19 pandemic. In 2020, malaria deaths increased by 10% compared with 2019, to an estimated 625,000. However, between 2021 and 2023, the number of deaths decreased to 597,000. Of these, 73.7% were children under the age of five—reflecting a reduction in mortality in this age group from 2000 to 2023. Malaria remains prevalent in tropical and subtropical regions, such as sub-Saharan Africa, Southeast Asia, Latin America, and the Eastern Mediterranean (World Health Organization, 2022; World Health Organization, 2024).

Malaria is an illness caused by protozoa belonging to the genus *Plasmodium.* These protozoa are part of the phylum Apicomplexa, which is known for having a specialized cellular organelle called the apical complex. This organelle is crucial for the invasion of plasmodia into the host cell (Rich and Ayala, 2003). There are more than 200 species of *Plasmodium*, of which five are known to infect humans: *Plasmodium falciparum*, *P. vivax*, *P. malariae*, *P. ovale*, and *P. knowlesi*. The last one typically infects non-human primates in Southeast Asia (Rich and Ayala, 2003), and among all the species, *P. falciparum* is the most prevalent malaria parasite worldwide, with Africa bearing the highest burden of the disease (95% of cases) (World Health Organization, 2022).

*Plasmodium falciparum* infection can progress to severe and lethal forms in non-immune people, while *P. vivax* is the most widely geographically distributed species and responsible for the majority of malaria cases in Americas, generally related to non-lethal infections (Rich and Ayala, 2003). There are four species of plasmodia parasites infecting rodents, which comprise valuable tools for studying malaria: *P. yoelii*, *P. berghei*, *P. chabaudi*, and *P. vinckei* (Rich and Ayala, 2003).

**Figure 1:**
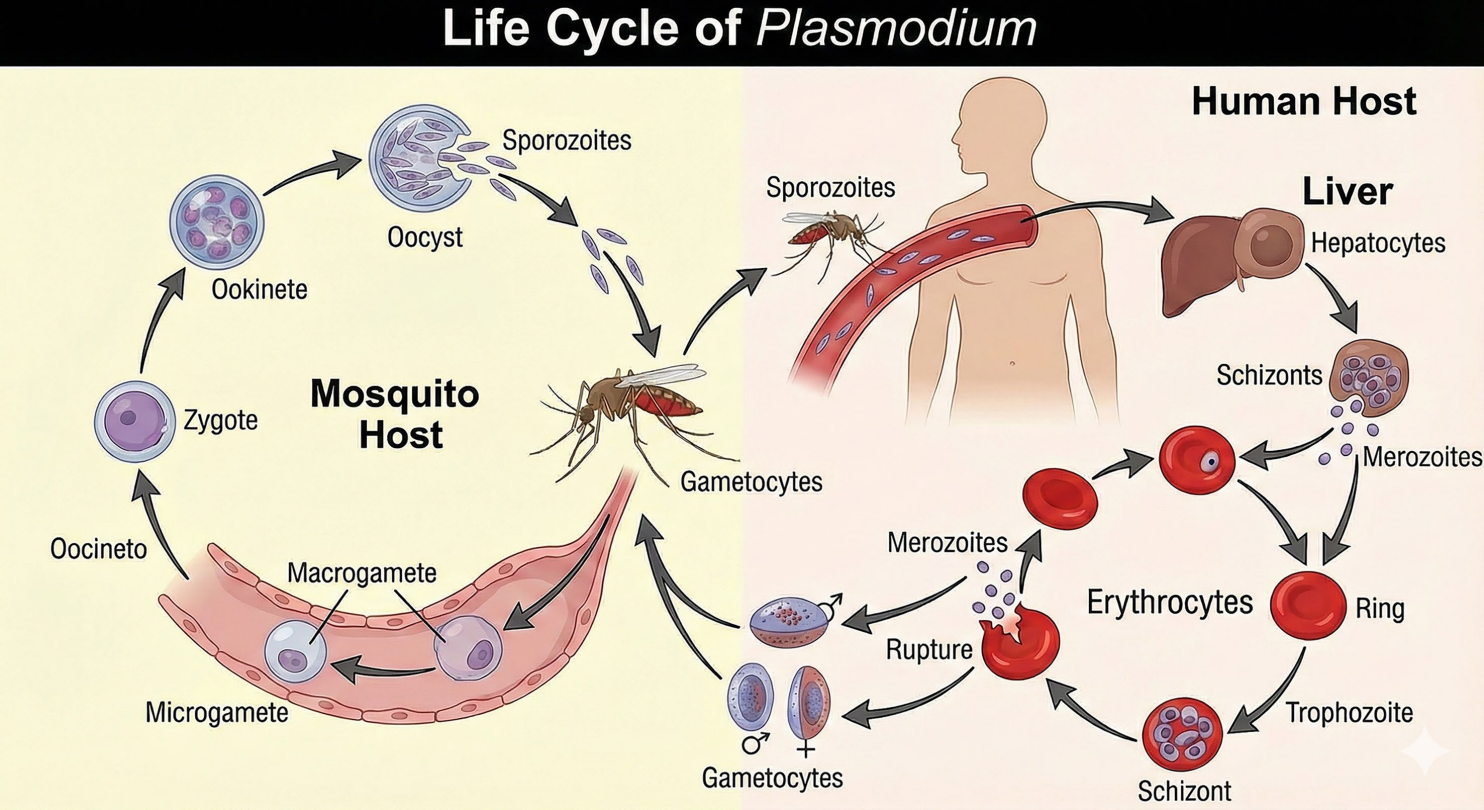
Life cycle of *P. falciparum* in the mosquito and human hosts. An infected *Anopheles* mosquito injects sporozoites into the human bloodstream during a blood meal. Sporozoites migrate to the liver, invade hepatocytes, and develop into hepatic schizonts, which release merozoites into the circulation. In the asexual blood stage, merozoites repeatedly invade erythrocytes and progress through ring, trophozoite, and schizont forms, causing rupture of red blood cells and clinical symptoms. A fraction of intra-erythrocytic parasites differentiates into male and female gametocytes, which are taken up by another mosquito. In the mosquito midgut, gametocytes develop into micro- and macrogametes, fertilize to form a zygote, develop into an ookinete and oocyst, and finally produce sporozoites that migrate to the salivary glands, completing the cycle. Image generated by AI (ChatGPT).

The life cycle of *Plasmodium* includes a sexual phase (sporogonic) in the *Anopheles* mosquito, the vector of the disease, and an asexual phase (schizogonic) in humans (Figure 1). The life cycle of the plasmodia in the human host begins when the female mosquito transfers haploid sporozoites into the skin while biting. After entering the bloodstream, the mobile sporozoites migrate to the liver and infect hepatocytes. In these cells, sporozoites multiply by schizogony, differentiating into schizonts full of thousands merozoites, the forms capable of infecting red blood cells, which will enter the bloodstream, beginning the blood or symptomatic phase of the disease (Cowman et al., 2016).

In the life cycles of *P. vivax* and *P. ovale*, not all sporozoites that infect the hepatocyte undergo schizogony. Instead, some forms remain in a state of latency in the hepatocyte, which is why they are called hypnozoites. These latent forms cause late relapses of the disease, which occur after weeks, months, or years, usually within six months for most *P. vivax* strains. Relapses are, therefore, new erythrocytic cycles resulting from late schizogony of dormant parasites within hepatocytes (Howes et al., 2016; Mueller et al., 2009).

In the blood stage, merozoites multiply, within red blood cells, also by schizogony, differentiating in three successive and morphologically distinct blood stages: the young trophozoite (ring forms), the mature trophozoite and, finally, the schizont. As the schizont matures, erythrocytic merozoites are formed and released in the blood after erythrocyte lysis, initiating a new stage of erythrocyte invasion. This completes the asexual cycle of the parasite. In this way, the parasite’s erythrocytic cycle is maintained, resulting in clinical symptoms of the disease (Cowman et al., 2016).

Instead of differentiating into schizonts, some merozoites develop into sexual erythrocytic forms known as gametocytes. During this stage, gametocytes differentiate into male (microgametocytes) and female (macrogametocytes), and these two forms can be distinguished by morphology (Taylor and Read, 1997; Meibalan and Marti, 2017). Once mature male and female gametocytes are taken up in a bloodmeal gametogenesis is stimulated by conditions in the mosquito midgut, including low temperature, high pH and the presence of xanthurenic acid (Billker et al., 1998).

The male gametocyte undergoes three rounds of DNA replication, resulting in a cell with eight nuclei, which generates flagellated male gametes by the process of exflagellation. The female gametocyte, on the other hand, does not undergo exflagellation, the female does round up and emerge from the RBC. The fusion of the male and female gametes results in the formation of a mobile diploid zygote (ookinete), which can take between 6 to 14 h to mature, depending on the species of *Plasmodium*. The ookinete migrates through the midgut epithelium, establishing itself beneath the basal membrane, where it undergoes sporogony.

This process involves a reductional meiotic division followed by several mitotic divisions that produce oocysts containing thousands of haploid sporozoites, which are subsequently released from the oocyst. The sporozoites travel to the salivary glands via the hemolymph, ensuring transmission to humans and maintenance of the parasitic cycle (Janse et al., 1988; Sinden, 1983).

The clinical symptoms of malaria are directly associated with the erythrocytic cycle of the disease, characterized by infection and lysis of red blood cells. The disease manifests itself in an asymptomatic to mild form, with symptoms such as fever, headache, nausea, drowsiness, among others. However, as the infection progresses, in the absence of prompt diagnosis and appropriate treatment, malaria may evolve into severe disease, most often characterized by severe anemia, cerebral malaria, and metabolic acidosis. Clinically significant severe malaria is largely confined to infections with *P. falciparum* and *P. knowlesi*; the other species tend to cause more self-limited illness, in part because they invade only a subset of red blood cells (Cowman et al., 2016).

In endemic areas, where infection and reinfection rates are high, severe and fatal forms of the disease especially affect children and immunosuppressed people, with the remainder of the population developing mild or asymptomatic forms of the disease (World Health Organization, 2024). This is explained by the slow and progressive development of immunity to the disease, promoted by continuous exposure to the parasite. However, malaria immunity is not sterile and, therefore does not prevent the re-establishment of new infections, only protecting against the acute symptoms of the disease. In this scenario, asymptomatic individuals are important reservoirs of gametocytes, contributing to the continuity of the cycle and transmission (Cowman et al., 2016). It is estimated that asymptomatic infections have 2.66 times greater odds of malaria transmission to mosquitoes than symptomatic infections (Sumner et al., 2021).

Strategies for controlling malaria include using insecticide-treated nets to control the vector and vaccines against pre-erythrocytic blood-stage (Keleta et al., 2021). Developing a vaccine against a complex eukaryotic parasite like *P. falciparum* is challenging. The current RTS, S vaccine, which targets the circumsporozoite surface protein (CSP), is only approved for use in children in high transmission areas (World Health Organization, 2022; Stoute et al., 1997; Neafsey et al., 2015). Resistance of *Anopheles* mosquitoes to different classes of insecticides has been reported in many part of the world, as well as chemoresistance of plasmodia to artemisinin-based combination therapies, which comprise the first-line treatment for *P. falciparum* (World Health Organization, 2022). Furthermore, most drugs do not eliminate gametocytes, except the 8 aminoquinolines, tafenoquine and primaquine (Ouologuem et al., 2022; Stone et al., 2022), which on the other hand cause hemolysis in G6PD deficiency (White, 2013).

Controlling malaria and plasmodia transmission is an ongoing challenge. Developing transmission-blocking vaccines is a promising strategy to combat this issue. Such vaccines can hinder the development of gametes by, for instance, promoting complement-mediated destruction of gametes (Duffy, 2021; Bansal and Kumar, 2024). Other approaches include induction of antibodies against antigens expressed in the mosquito midgut, or against antigens expressed on the surface of gametocyte-infected red blood cells (Chawla et al., 2021; Alemayehu, 2023; Saeed et al., 2008; Armistead et al., 2014). To achieve this, understanding gametocytogenesis is crucial.

**Figure 2:**
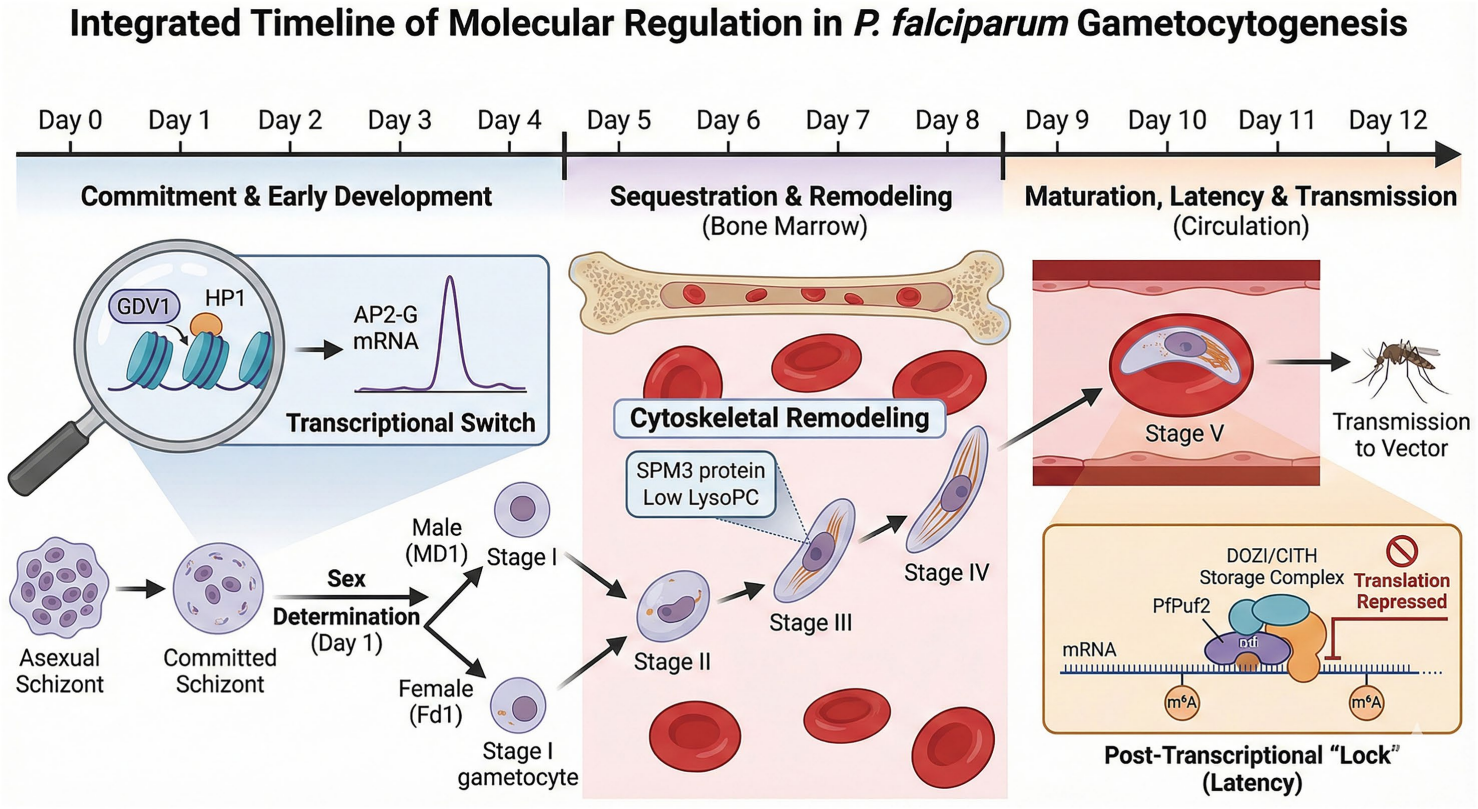
Integrated timeline of molecular regulation during *P. falciparum* gametocytogenesis. The development from a sexually committed schizont to a mature Stage V gametocyte spans approximately 12 days. (Phase I) Commitment (Day 0–2): The epigenetic removal of H3K9me3/HP1 by GDV1 allows the expression of the master transcription factor AP2-G, initiating sexual differentiation. (Phase II) Sequestration & remodeling (Day 3–9): Developing gametocytes (Stages I–IV) sequester in the bone marrow. During this period, cytoskeletal remodeling (involving proteins like SPM3) drives the morphological change from round to falciform shapes. (Phase III) Maturation & latency (Day 10–12): Mature Stage V gametocytes re-enter circulation. At this stage, post-transcriptional mechanisms become dominant: RNA-binding proteins (PfPuf2, DOZI/CITH) and epitranscriptomic marks (m^6^A) repress the translation of essential transcripts, maintaining the parasite in a latent, transmissible state until ingestion by789 the mosquito vector. Image generated by AI (Gemini).

While in the most virulent human malaria, *P. falciparum*, gametocytogenesis involves extensive morphological remodeling across five stages (I–V), this review focuses specifically on the molecular ‘switches’ of sexual commitment (transcriptional/epigenetic control) and the latency mechanisms (post-transcriptional/epitranscriptomic regulation) that prepare the mature gametocyte for transmission. We integrate these molecular checkpoints into a developmental timeline (Figure 2) to highlight novel therapeutic vulnerabilities.

## Gametocytogenesis

Gametocytogenesis is a crucial stage in the *Plasmodium* life cycle, necessary for transmission to vector mosquitoes. During each schizogony cycle, only about 2% of merozoites differentiate into gametocytes. Environmental stress can modulate gametocytogenesis both *in vitro* and *in vivo*. The number of sexually committed schizonts varies, but all merozoites arising from a given committed schizont are themselves sexually committed (Alemayehu, 2023). Sexual commitment varies from nothing to more than 10% *in vitro* and the field (Usui et al., 2019; Stewart et al., 2022).

Commitment to sexual development occurs in the canonical way, that is during the schizogony, each developing merozoite will either undergo sexual development or continue asexual multiplication after exit and reinvasion (next cycle conversion) (Bruce et al., 1990). However, under experimental conditions, sexual commitment may occur during very early ring stages, enabling parasites to develop directly into gametocytes without first undergoing schizogony (same-cycle conversion). To date, this type of same-cycle conversion has not been observed *in vivo* in response to environmental factors (Bancells et al., 2019).

**Figure 3:**
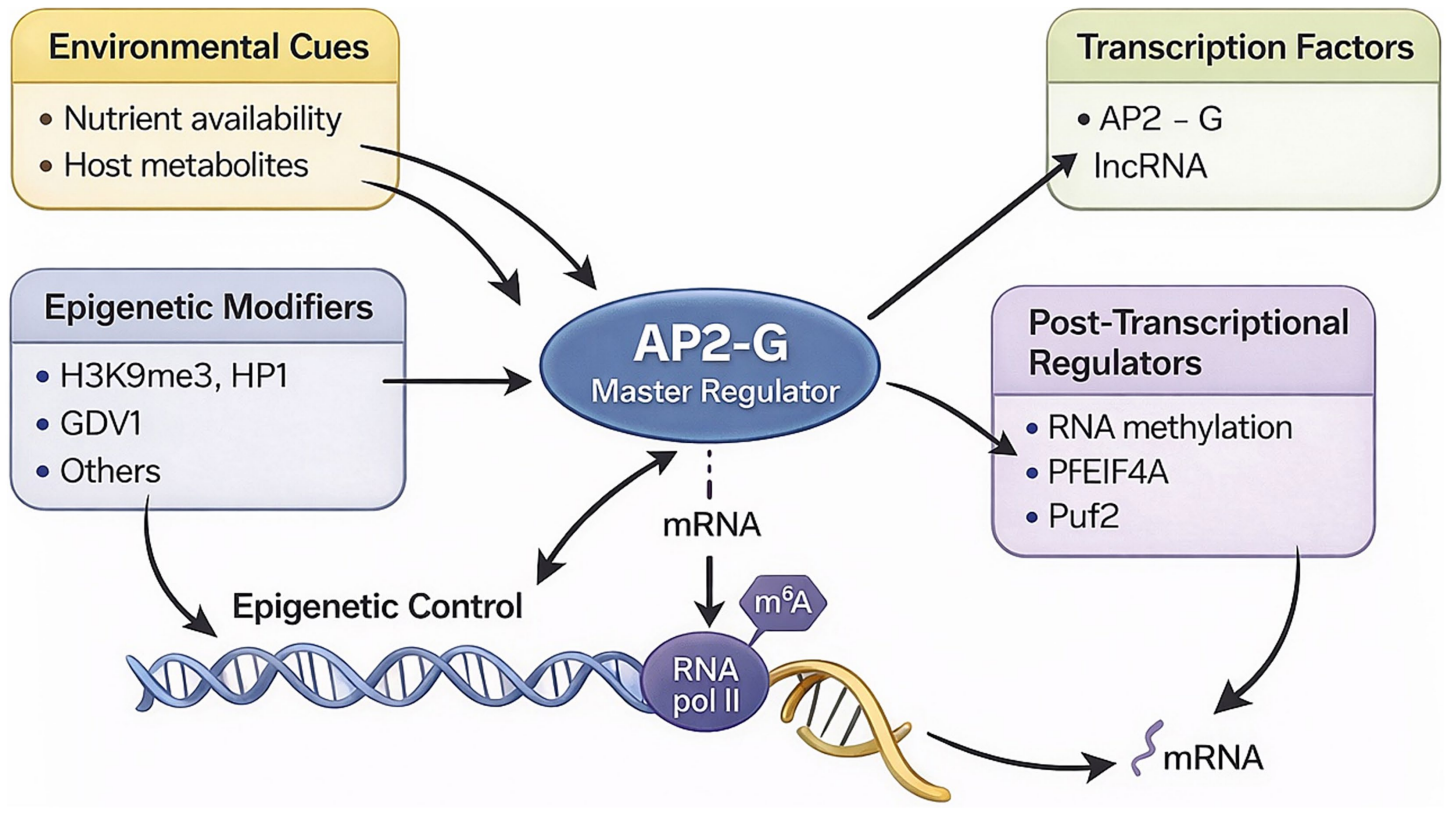
Regulatory pathways controlling AP2-G–dependent gametocytogenesis in *P. falciparum*. Schematic overview of how distinct regulatory layers converge on the master regulator AP2-G. Environmental cues (nutrient availability and host metabolites) signal through epigenetic modifiers (H3K9me3/HP1, GDV1 and others) and transcriptional components including AP2-G and associated lncRNAs. These pathways activate AP2-G, which drives a gametocyte-specific transcriptional program. Post-transcriptional regulators (RNA methylation, PfEIF4A, Puf2 and related RNA-binding proteins) further modulate mRNA processing, stability, and translation, integrating chromatin, transcriptional, and RNA-based control of sexual development. Image generated by AI (ChatGPT).

Gametocytogenesis can be enhanced under various *in vitro* conditions, such as using conditioned medium or adding red blood cell lysate and lymphocytes together with serum (Keleta et al., 2021; Smalley and Brown, 1981). However, this induction is not required for sexual commitment. Culture conditions can change the proportion of parasites that commit, but these factors are not obligatory triggers, unlike the drop-in temperature and low pH/xanthurenic acid, which are required to initiate gametogenesis (Usui and Williamson, 2021). *In vivo* gametocytogenesis has been related to high parasitemia, drug treatment, anemia, and host immune response, many of these conditions are consistent with prolonged infection (Bruce et al., 1990; Nacher et al., 2002; Bousema et al., 2006; Looareesuwan et al., 2023; Drakeley et al., 2006; Bousema et al., 2010; Andolina et al., 2021). In addition to environmental stresses, genetic and epigenetic factors determine the commitment of merozoites, initiating differentiation into gametocytes (Josling and Llinás, 2015; Brancucci et al., 2014) (Figure 3).

Regarding drug treatments, several studies have correlated an increase in gametocytes with the use of antimalarials, such as sulphadoxine-pyrimethamine (Sokhna et al., 2001; Fehintola et al., 2012; Sowunmi et al., 2006; Buckling et al., 1999; Talman et al., 2004), in both *in vivo* and/or *in vitro* assays, showing a strong association with resistance to these drugs (Barnes and White, 2005). Although artemisinin-based therapies have demonstrated gametocytocidal activity (Dutta et al., 1989; Snider and Weathers, 2021; Ippolito et al., 2017), this effect is limited against mature gametocytes (Mehra and Bhasin, 1993; Kumar and Zheng, 1990). Moreover, there has recently been an increasing number of reports of artemisinin resistance emerging in countries across the African and Asian (Mairet-Khedim et al., 2021; Pandey and Pandey-Rai, 2016; Hamilton et al., 2019). In addition to artemisinin, primaquine has proven effective against gametocytes; however, as mentioned previously, its use is limited by its high toxicity, which can induce hemolytic anaemia in G6PD-deficient individuals (White, 2013; Vale et al., 2009).

Furthermore, studies on *P. vivax* have demonstrated that low doses of primaquine are ineffective at fully blocking transmission to the mosquito vector (Getachew et al., 2025). Peatey et al. (2009) reported an increase in the number of gametocytes during *in vitro* treatment of *P. falciparum* cultures with low concentrations of primaquine, which may result either from enhanced gametocytogenesis or from the maturation and post-treatment survival of pre-existing gametocytes. These findings suggest that effective treatment with full therapeutic doses should not increase gametocyte carriage; however, they also indicate that, in cases of resistance, and given that primaquine is a long-acting drug, blood levels may fall to subtherapeutic concentrations, potentially increasing gametocyte carriage and parasite transmission (Peatey et al., 2009). These points reinforce the need to discover new antimalarial compounds, particularly those that target the sexual stages of the parasite, in order to more effectively block parasite transmission to mosquito vectors.

The complete development of *P. falciparum* gametocytes take approximately 10–12 days (Figure 2), longer than any other species of *Plasmodium* (Josling and Llinás, 2015; Gautret et al., 1999). The process of gametocytogenesis occurs in five stages (I-V) that are morphologically distinct and visible under a microscope for *P. falciparum.* However, it can be challenging to accurately identify gametocytes of other species based solely on morphology (Cowman et al., 2017). Stage I–IV *P. falciparum* gametocytes sequester in extravascular niches of hematopoietic tissues, such as the bone marrow and spleen, whereas mature stage V gametocytes re-enter the peripheral bloodstream, where they can be taken up by an Anopheles mosquito during a blood meal (Gardiner and Trenholme, 2015; Joice et al., 2014; Venugopal et al., 2020).

Initially, in *P. falciparum,* gametocytes are similar in appearance to asexual trophozoites, with a round shape and no noticeable membrane changes. However, upon reaching stage II of maturation, gametocytes elongate and take on the characteristic teardrop shape, making them easily distinguishable (Hawking et al., 1971). Throughout this process, the shape of the gametocyte becomes a half-moon and can occupy up to half of the area of the red blood cell. In stage III, the cells continue to elongate and form a slightly distorted diamond shape (Talman et al., 2004).

In the fourth stage of gametocytes, they elongate and become thin, growing towards their central axis. In this stage, there are many mitochondria, ribosomes, and osmophilic bodies. Gametocytes become morphologically mature in the fifth stage (V), and sexual dimorphism is present, and a well-developed subpellicular membrane is evident. Females have a more elongated and curved shape with a relatively small nucleus containing nucleolus and concentrated pigment, while males have a thicker shape with a larger nucleus containing diffuse pigment lacking nucleolus (Talman et al., 2004).

While the morphological differentiation into microgametocytes and macrogametocytes is well characterized, the gametocyte sex ratio is a dynamic trait essential for transmission efficiency. Typically, *P. falciparum* infections exhibit a female-biased sex ratio, optimizing reproductive output since a single male can produce up to eight microgametes capable of fertilizing multiple females. However, the parasite can adaptively alter this ratio in response to environmental cues, such as host stress, drug pressure, or low gametocyte density (Henry et al., 2019). Under these challenging conditions, increasing the proportion of males serves as a “fertility insurance” strategy, maximizing the probability that at least one male gametocyte will be ingested by the mosquito to ensure fertilization (Tadesse et al., 2019). Consequently, understanding the molecular regulation of sex allocation is vital for the development of effective transmission-blocking strategies.

## Transcriptional regulation of sexual commitment

Recent studies have identified transcriptional regulators for early gametocyte development and sexual differentiation in *P. falciparum*. Single-cell transcriptomic analyses revealed distinct developmental trajectories and sex-specific transcriptional programs that are established early during sexual commitment, contributing to the emergence of morphologically distinct male and female gametocytes (Dogga et al., 2024; Gomes et al., 2022).

Elucidating the transcriptional regulation controlling sexual differentiation is key to understanding the molecular basis of malaria transmission to mosquito (Cowman et al., 2017). The study by Balaji et al. (2005) identifies a large family of plant-like AP2-domain transcription factors (ApiAP2) in Apicomplexan parasites and proposes them as key regulators of their stage-specific gene expression programs. Several transcription factors (TFs) from the Apetala 2 (AP2) family are involved in the mechanisms regulating gametocytogenesis and, currently, it is believed that AP2-G transcription factor plays a crucial role in gametocytogenesis regulation (Josling et al., 2018; Kafsack et al., 2014; Sinha et al., 2014).

AP2-G features a single DNA-binding domain that recognizes a specific DNA sequence present in the promoters of various gametocyte differentiation genes and by using reporter genes, it is suggested that AP2-G is an activator of transcription (Josling et al., 2018; Kafsack et al., 2014; Sinha et al., 2014). AP2-G was also identified as the target of mutations in gametocyte-deficient parasite lines in both *P. falciparum* and *P. berghei* (Kafsack et al., 2014; Sinha et al., 2014). During the asexual replication cycle, AP2-G expression is silenced. Once AP2-G transcription is induced and the levels of AP2-G protein increase, it enhances its own transcription (Kafsack et al., 2014; Sinha et al., 2014; Poran et al., 2017) and initiates gametocytogenesis through a cascade of gene expression that is tightly controlled through complex regulatory systems (Josling et al., 2018; Poran et al., 2017). In this way, AP2-G plays a direct role in orchestrating transcription not just during commitment but also into stage I of gametocytogenesis. A more recent study using the DNA ChIP-sec technique showed that the expression of gametocyte differentiation marker genes, including *pfs16, pfg27, pfg14.744, pfg14.748, etramp10.3 and gexp05* (Kafsack et al., 2014), are directly regulated by AP2-G (Josling et al., 2020).

Epigenetic regulation is likely responsible for the control of AP2-G expression, through the formation of reversible chromatin structures. In asexual blood stage cultures, the *ap2-g* locus is typically found in a silenced heterochromatic state in most parasites, and a permissive transcriptional state may only occur in a few sexually committed parasites. In *P. falciparum*, *ap2-g* is not expressed in most cells because it is epigenetically silenced by H3K9me3 (trimethylation of lysine 9 on histone H3), heterochromatin protein 1 (HP1) (Brancucci et al., 2014), and histone deacetylase 2 (Hda2) (Coleman et al., 2014).

Furthermore, in a transcriptional analysis of *P. falciparum*, the gametocyte development 1 gene (GDV1), which encodes a perinuclear protein, was identified as a regulator of early gametocyte development (Eksi et al., 2012; Filarsky et al., 2018). Deletions in its coding locus have been observed in gametocyte-deficient strains, whereas in gametocyte-producing strains, this same region remained intact (Eksi et al., 2012; Filarsky et al., 2018). The study by Filarsky et al. (2018) analyzed the interaction between the GDV1 gene and the HP1 gene. This interaction results in the eviction of HP1 from chromatin, thereby lifting the repression of ap2-g and other gametocyte-specific genes. Conditional overexpression of GDV1 led to a significant increase in the rate of sexual conversion, and transcriptomic analyses confirmed the induction of gametocyte-related transcriptional programs following GDV1 activation (Eksi et al., 2012).

Lysophosphatidylcholine (LysoPC) has been identified as a modulator of gametocytogenesis (Brancucci et al., 2017). LysoPC is a common component of human serum that the parasite uses as a key substrate for membrane phospholipid metabolism. RNA-seq analyses revealed a dramatic induction of *ap2-g* transcription upon LysoPC depletion, leading to increased gametocyte production *in vitro* and indicating that this lipid acts upstream of this master regulator of gametocytogenesis (Brancucci et al., 2017). LysoPC depletion triggers metabolic changes that may influence the induction of epigenetic factors, such as chromatin-modifying enzymes, resulting in *ap2-g* activation and commitment to sexual differentiation. A reduction in LysoPC levels was the first defined environmental signal shown to act as an enhancer of gametocyte production. Consequently, host environments with reduced LysoPC levels, such as the bone marrow, are suggested to be more favorable for gametocyte proliferation (Brancucci et al., 2017; Prajapati et al., 2020).

## Transcriptional regulation of gametocytogenesis

Following AP2-G-mediated commitment to gametocytogenesis the parasite must then undergo morphological and sexual differentitation. Recent work indicates that a transcriptional regulator HDP1 (homeodomain protein 1) is induced after AP2-G induced commitment as part of an early regulatory wave that implements the morphogenetic program required for stage I–II progression (Campelo Morillo et al., 2022). In line with this role, HDP1 localizes to the gametocyte nucleus and is strongly chromatin-associated, and its loss results in an early, lethal developmental arrest characterized by failure of IMC expansion and impaired acquisition of the elongated gametocyte morphology (Campelo Morillo et al., 2022).

The parasite must also determine its sexual identity following AP2-G–mediated commitment to gametocytogenesis. Recently, the gene md1 (male development 1) was identified as the master regulator of this process, being both necessary and sufficient to drive the male fate. This determination mechanism involves a bistable transcriptional switch at the md1 locus: high expression of the MD1 protein directs the parasite toward the male phenotype, whereas its repression results in female development. MD1 functions through two distinct domains, with its C-terminal LOTUS domain being critical for regulating the specific gene-expression program required for male differentiation, while the N-terminal region is required for male sex determination (Gomes et al., 2022).

Parallel studies in the rodent malaria model *P. berghei* have further elucidated this sex-determination logic, identifying not only the male-determining factor (PbMd1) but also a female-determining factor (PbFd1). In this system, sex determination relies on the reciprocal regulation between these two genes: PbMd1 drives male development while repressing PbFd1, whereas PbFd1 is essential for female differentiation. Crucially, the *P. falciparum* homolog of PbMd1 is the previously described MD1, which performs the same male-determining function. Although a syntenic homolog of PbFd1 exists in *P. falciparum* (PF3D7_1361800), its functional role appears to be less dominant or more complex than in the murine model, suggesting that, while the core machinery is conserved, species-specific regulatory nuances may exist in the interplay between these sex-determining factors (Russell et al., 2023).

Some studies also suggest the putative involvement of non-coding RNA (ncRNA) regions that do not contribute to protein formation but play a key role in chromatin condensation and, thus, in gene expression regulation (Reyser et al., 2024; Batugedara et al., 2023). Among them are long non-coding RNAs (lncRNAs), which can interact with DNA, RNA, and proteins, assisting in transcriptional regulation, promoter accessibility, and even chromatin-remodeling enzyme activity (Broadbent et al., 2011). During gametocytogenesis, specific lncRNA molecules gdv1-lncRNA and md1-lncRNA were detected, playing partial roles in sexual differentiation and determination (Filarsky et al., 2018). The gdv1-as-lncRNA, an antisense transcript of the GDV1 gene (i.e., transcribed in the reverse direction), interferes with GDV1’s ability to initiate cell differentiation. When gdv1-as-lncRNA is expressed, GDV1 cannot induce the onset of cellular differentiation (Filarsky et al., 2018).

A recent study by Wichers-Misterek et al. (2023) reported the identification of a new microtubule-associated protein (MAP), designated SPM3, which is conserved in *Plasmodium*. MAPs constitute a group of proteins that bind to microtubules and perform functions such as microtubule stabilization, anchoring of organelles and membranes, and regulation of other proteins (Bodakuntla et al., 2019). Disruption of the PfSPM3 gene in *P. falciparum* resulted in the absence of gametocytes with their characteristic falciform shape, instead producing rounded gametocytes with disorganized subpellicular microtubules (SPMTs), as observed by live-cell microscopy (Wichers-Misterek et al., 2023).

The authors then conducted experiments using the rodent malaria species *P. berghei*, exposing rodents infected with the knockout parasite to the mosquito vector, and found that gametocyte and ookinete development, as well as midgut traversal, were not affected by disruption of the PbSPM3 gene. Conversely, sporozoite motility was impaired, resulting in reduced numbers of parasites in the mosquito midgut and, consequently, diminished transmission to the vertebrate host. This difference may, at least in part, reflect the distinct gametocyte and sporozoite morphologies of *P. berghei* compared with *P. falciparum*, as species-specific structural features could influence cytoskeletal organization, parasite–host interactions, and stage-specific requirements for SPM3 function. Examination of SPMT organization in these sporozoites revealed an increased distance between SPMTs and the inner membrane complex (IMC) in SPM3-knockout parasites compared with the wild type, suggesting an anchoring role for SPM3 in connecting SPMTs to the IMC during this life-cycle stage (Wichers-Misterek et al., 2023).

## Post-transcriptional regulation of gametocytogenesis

*Plasmodium falciparum* transcriptome profiling reveals that most genes exhibit a specific temporal pattern of activity during the intraerythrocytic developmental cycle, suggesting that *Plasmodium* species use gene-specific transcriptional activation and suppression to produce transcripts only when their gene products are necessary (Otto et al., 2010). It is worth noting that while there are similarities between the transcript and protein abundance profiles, the raw mRNA and protein levels are not always directly correlated (Foth et al., 2008; Foth et al., 2011). Studies have shown that post-transcriptional mechanisms play a significant role in regulating protein expression in *P. falciparum*. In gametocytes and salivary gland sporozoites, the release of mRNA from translational repression enables rapid adaptation following a host switch (Mair et al., 2010).

The study by Painter et al. (2017) demonstrated that post-transcriptional regulation is also critical for gametocyte development. Their data showed that the DNA-binding motif of AP2-G is associated with several mRNAs transcribed in sexually committed parasites. They use 4-TU labelling with FCU-GFP to separate newly transcribed mRNAs from stabilized (pre-existing) mRNAs and, via the pfs16 promoter, mainly capture sexual commitment and early Stage I gametocytes. The central finding is that entry into the sexual pathway depends on transcriptional reprogramming plus selective mRNA stabilization. The dynamics of mRNA involved in gametocytogenesis commitment rely on a balance between transcription and stabilization in gametocyte-producing lines. The absence of this transcript stabilization, possibly due to the lack of an RNA-binding protein, may result in the parasite’s inability to commit to gametocytogenesis. Therefore, the stabilization of gametocyte transcripts by RNA-binding proteins is likely a key mechanism regulating the early stages of gametocyte development (Painter et al., 2017; Painter et al., 2018).

Two members of the Puf family of RNA-binding proteins have been characterized in *Plasmodium*, PfPuf1 and PfPuf2, both of which are involved in the regulation of gametocytogenesis. Available data indicate that Puf proteins are expressed early during gametocytogenesis, with transcripts detectable in committed ring stages and early gametocytes, supporting a role in regulating gene expression before late-stage maturation (Cui et al., 2002; Fan et al., 2004). Puf proteins are found across a wide range of organisms and generally act as translational repressors by binding to transcripts and preventing their translation. In *P. falciparum*, disruption of PfPuf2 leads to an increase in gametocytogenesis, while more recently it has been shown that in the absence of PfPuf1, gametocyte production decreases (Miao et al., 2010; Shrestha et al., 2016). Although the precise mechanism by which PfPuf1 regulates gametocytogenesis has not yet been elucidated, it is likely that, as a member of the Puf protein family, it also functions as a repressor of sexual development (Josling et al., 2018).

Another important cytoplasmatic component involved on post-transcriptional regulation are the ribonucleoproteins (RNPs), a complex formed by RNA and proteins commonly involved in functions of gene expression regulation, RNA processing and proteins synthesis. The DDX6 family of DEAD-box RNA helicases are components of messenger ribonucleoprotein (mRNP) complexes in humans, involved in translational regulation, mRNA storage, and degradation, as well as in the assembly and modulation of P-body homeostasis (Di Stefano et al., 2019; Weston and Sommerville, 2006). Processing-bodies (P-bodies), a specific type of RNP, are cytoplasmic, membrane-less granules present in eukaryotic cells that regulate messenger RNA (mRNA) metabolism. In most eukaryotes, they are involved in mRNA storage, degradation, and translational repression, playing a key role in post-transcriptional gene regulation (Luo et al., 2018).

However, although recent studies have shown that certain mRNPs in *P. falciparum* are associated with proteins typically linked to mRNA decay (Min et al., 2024), functional evidence indicates that these complexes primarily mediate long-term translational repression and mRNA stabilization, rather than acting as constitutive mRNA degradation hubs, having an essential function during sexual stages and for parasite development in the mosquito vector (Cui et al., 2015). Furthermore, to date, studies with other *Plasmodium* species have not identified RNA turnover components (Mair et al., 2010).

In several organisms, homologues of DDX6 is implicated in mRNA silencing and storage, particularly for transcripts encoding proteins associated with meiotic progression (Weston and Sommerville, 2006). A homolog of this RNA helicase was identified in *P. berghei*, known as DOZI (Development of Zygote Inhibited), which exhibits activity similar to DDX6. DOZI functions by storing many RNA transcripts, including p25 and p28 transcripts, in female gametocytes, which are precursors of female gametes, for later use after gametogenesis (Mair et al., 2006). These transcripts encode proteins involved in zygote development within the mosquito midgut (Kumar and Carter, 1985). Disruption of *pbdozi* diverted the transcripts toward degradation pathways by inhibiting the formation and destabilizing mRNP complexes associated with p25 and p28 mRNAs (Mair et al., 2006).

In *P. falciparum*, disruption of *pfdozi* resulted in increased asexual proliferation, but led to a reduction in sexual commitment. However, the phenotype is not solely explained by a decrease in commitment, as sexually committed parasites can still be detected. Instead, *pfdozi* disruption also causes a pronounced defect in post-commitment gametocyte development. Genetically modified parasite lines were able to develop gametocytes only up to stage III, after which development was arrested and gametocyte death occurred, indicating a critical role for DOZI in gametocyte maturation beyond early stages. Although pfdozi is expressed at significantly higher levels in female gametocytes, both male and female gametocytes were affected. DOZI is expressed early during gametocytogenesis, beginning in sexually committed parasites and early gametocytes, consistent with its function in post-transcriptional regulation required for later differentiation. At the cellular level, a marked reduction in microtubules and inner membrane complex (IMC) structures was observed, accompanied by an increase in disorganized cytoskeletal elements, providing a mechanistic explanation for the failure of gametocyte elongation and maturation (Min et al., 2024).

Transcriptomic profiling of stage III gametocytes showed that, relative to WT 3D7, Δpfdozi (clone K2) displays widespread transcript loss: ~80% of the downregulated mRNAs are gametocyte and/or ookinete-enriched, including AP2-G transcription factor, LCCL-domain proteins, and markers such as pfs25 and pfs28. Even though gametocytogenesis can be induced in wild-type parasite cultures under nutritional stress (e.g., by retaining the culture medium) (Fivelman et al., 2007), the expression levels of early gametocyte genes were among the most severely reduced (Brancucci et al., 2017; Min et al., 2024).

During gametocyte differentiation, PfDOZI is linked to a shift in mRNA fate: erythrocyte-invasion transcripts show enhanced turnover, whereas morphogenesis-related transcripts are preferentially maintained. PfDOZI associates with P-body–like assemblies containing decapping/deadenylation machinery, and Δpfdozi parasites display prolonged half-lives for a subset of transcripts after transcriptional shutoff—supporting an indirect role for PfDOZI in promoting mRNA decay. In later sexual development, DOZI participates in gametocyte mRNP complexes that store and translationally repress female mRNAs for subsequent expression in mosquito stages (e.g., the ookinete), resembling P-granule–like structures described in other organisms (Min et al., 2024).

In another study by Mair et al. (2010), with *P. berghei*, immunoprecipitation assays revealed that DOZI forms a complex with 16 proteins, including homologs of worm CAR-I and fly Trailer Hitch (CITH). The same repressed transcripts mentioned earlier were also co-eluted with CITH. The DOZI/CITH mRNP complex includes additional proteins homologous to P-granule components found in metazoans, such as 5′ cap-binding factor eIF4E, poly(A)-binding protein (PABP), the BRUNO homolog (HoBo), and the Musashi homolog (HoMu). Among them, eIF4E serves as the 5′ cap-binding factor and plays a critical role in translation initiation; within the mRNP context, it contributes to the selective sequestration of mRNAs, thereby preventing their untimely translation. PABP, the poly(A)-binding protein, interacts with the polyadenylated tails of mRNAs to promote their stability and facilitate translational control. The BRUNO homolog (HoBo) functions as a translational repressor, binding to specific mRNAs to inhibit their translation until proper developmental cues are received. Similarly, the Musashi homolog (HoMu) represses the translation of target mRNAs, particularly those involved in cell fate determination, and is implicated in regulating gametocyte development in the parasite. Together, these proteins form a post-transcriptional regulatory hub that governs the timing and localization of gene expression, ensuring the coordinated progression of sexual differentiation and transmission readiness in *Plasmodium* (Anderson and Kedersha, 2008; Chekulaeva et al., 2006; Kawahara et al., 2008).

Therefore, these different configurations of DOZI-containing complexes indicate that DOZI function is highly dependent on its proteomic context. In summary, the DOZI/CITH complex acts as a storage platform for the p25 and p28 transcripts, which are essential for parasite survival in the hostile environment of the mosquito midgut and for development of the earliest stages in the invertebrate host. This complex is present in developing female gametocytes, such that transcription and storage of these mRNAs in the vertebrate host enable their rapid availability and immediate translation following mosquito ingestion. Importantly, under natural conditions, p25 and p28 transcripts are stably maintained and translated at the appropriate developmental stage. In contrast, genetic disruption of the DOZI/CITH complex does not primarily result in translational derepression but instead leads to destabilization and degradation of these mRNAs. Thus, loss of this complex experimentally abolishes mRNA storage, preventing zygote differentiation into ookinetes. The requirement for DOZI/CITH therefore reflects a regulated storage mechanism rather than a naturally occurring pathway of transcript turnover (Anderson and Kedersha, 2008; Chekulaeva et al., 2006; Kawahara et al., 2008).

Another key finding was the association of ALBA proteins (Acetylation Lowers Binding Affinity) with the female DOZI/CITH complex (Mair et al., 2010; Min et al., 2024; Mair et al., 2006). ALBA family proteins are small, highly conserved factors involved in chromatin maintenance and transcriptional repression. In archaea, ALBA functions by organizing and regulating the genome via DNA packaging, cooperative binding, dimerization/oligomerization, and transcriptional repression through acetylation/deacetylation, possibly even acting as sequence-specific transcription factors. In eukaryotes, ALBA proteins participate in translational regulation, binding different RNA species, repressing translation, and stabilizing and protecting transcripts (Goyal et al., 2016).

Among the ALBA family, ALBA4 is specific to the Apicomplexa lineage, is highly conserved, and consistently found bounding to both DNA and RNA. In *P. berghei*, ALBA1–4 proteins have been shown to associate with DOZI and CITH in transcriptionally repressive complexes (Mair et al., 2010). Within the DOZI/CITH/ALBA complex, ALBA proteins mediate the recognition of specific transcripts and orchestrate the ordered release of each repressed mRNA. The function of this complex is specifically linked to selective translational repression, with DOZI and CITH being essential for zygote and female gametocyte development, whereas ALBA4 contributes to the regulation of male gametocyte activation and to the coordinated development of sporozoites in the mosquito (Mair et al., 2010).

A study of ALBA4 in *P. yoelii* demonstrated PyALBA4 as a regulator of mRNA homeostasis across different stages of the parasite life cycle (Muñoz et al., 2017). In gametocytes, it represses premature activation of male sexual forms. PyALBA4 interacts with CDPK4, a calcium-dependent kinase essential for male gamete exflagellation, and its disruption leads to increased motility centers (Muñoz et al., 2017). Although gene repression led to a higher number of activated male gametes, it did not affect the sex ratio (male-to-female gametocyte proportion), nor total gametocytemia, suggesting that ALBA4 is more directly involved in gametocyte activation rather than formation. The ALBA4-associated protein complex contributes to the stabilization and translation of specific transcripts, which are essential not only for mosquito-stage transmission and infection, but also for intrinsic gametocyte functions, such as calcium-dependent activities and components of the inner membrane complex (Muñoz et al., 2017) (Table 1).

**Table 1:** Key post-transcriptional regulators with demonstrated functional roles in *Plasmodium*.

Protein	Species	Annotation	Demonstrated functional role	Ref(s)
AP2-G	*P. falciparum*	ApiAP2 transcription factor (DNA-binding motif)	The AP2-G DNA-binding motif is associated with mRNAs expressed in sexually committed parasites, linking commitment to post-transcriptional regulation.	Foth et al. (2011) and Mair et al. (2010)
PfPuf2	*P. falciparum*	PUF-family RNA-binding protein (translational repressor)	PfPuf2 disruption increases gametocytogenesis.	Painter et al. (2017), Painter et al. (2018), and Cui et al. (2002)
PfPuf1	*P. falciparum*	PUF-family RNA-binding protein (translational repressor)	Loss of PfPuf1 decreases gametocyte production.	Painter et al. (2017), Painter et al. (2018), and Fan et al. (2004)
DOZI	*P. berghei*	DDX6 homolog; mRNP/granule-associated factor	Stores and translationally represses maternal mRNAs (e.g., p25/p28) in female gametocytes; pbdozi disruption destabilizes mRNPs and diverts these transcripts to degradation, blocking post-fertilization development.	Weston and Sommerville (2006) and Ippolito et al. (2017)
PfDOZI (pfdozi)	*P. falciparum*	DOZI homolog; associates with P-body–like complexes	KO increases asexual proliferation, reduces sexual commitment, and arrests gametocytes at stage III with structural defects; a subset of transcripts shows prolonged half-lives after transcriptional shutoff (consistent with an indirect role in mRNA turnover).	Luo et al. (2018) and Brancucci et al. (2017)
CITH	*Plasmodium*	DOZI-complex component (Trailer Hitch homolog)	Forms a complex with DOZI; absence of the DOZI/CITH complex blocks zygote-to ookinete differentiation due to failure of appropriate mRNA repression/storage.	Foth et al. (2008), Cui et al. (2015), Mair et al. (2006), and Kumar and Carter (1985)
ALBA4	*P. yoelii (PyALBA4)*	ALBA-family RNA-binding protein (mRNA homeostasis/translation control)	Regulates mRNA homeostasis and represses premature activation of male sexual forms; interacts with CDPK4; disruption increases motility centers.	Anderson and Kedersha (2008)
PfCDPK4	*P. falciparum*	Ca^2+^-dependent protein kinase	Essential for male gametogenesis (exflagellation); KO blocks microgamete formation and transmission; central to a signalling network impacting translational control.	Chekulaeva et al. (2006)

This table summarizes proteins for which direct experimental evidence (e.g., genetic disruption and/or complex membership with phenotypic impact) supports roles in the post-transcriptional regulation of sexual commitment, gametocyte development, and maternal mRNA storage/translation; reference numbers correspond to those cited in the manuscript.

Functional studies have established PfCDPK4 as a key regulator of male gametogenesis in *P. falciparum* (Billker et al., 2004; Kumar et al., 2021). Parasites lacking this kinase fail to complete male development: exflagellation is arrested, functional microgametes are not produced and, as a consequence, transmission to the mosquito vector is severely compromised (Billker et al., 2004; Kumar et al., 2021). Phosphoproteomic profiling further revealed that PfCDPK4 sits at the center of an extensive signalling network, influencing phosphorylation events linked to DNA synthesis, protein translation and motility-associated processes that are all essential for successful male gamete formation (Kumar et al., 2021). Beyond its role in gametocytes, PfCDPK4 is also detected in early asexual blood stages, throughout gametocyte maturation and in sporozoites, where it predominantly localizes to the parasite membrane, and there is evidence suggesting a possible contribution to hepatocyte invasion (Kumar et al., 2021; Brochet et al., 2014). Nevertheless, among the multiple stages in which it is expressed, its most critical function appears to lie in orchestrating male gamete development. Consistent with this, PfCDPK4 targets several proteins involved in translational control, including CCR4–NOT1, the elongation factor eEF1A and the initiation factors eIF3B and eIF4G, highlighting its broader impact on the regulation of mRNA translation (Kumar et al., 2021).

## Post-transcriptional epigenetic modifications

Sexual differentiation in *P. falciparum* relies on the coordinated action of multiple epigenetic regulatory layers. Recent studies using single-cell transcriptomics and chromatin profiling have shown that early gametocytogenesis is accompanied by extensive chromatin remodeling, which contributes to the establishment of sex-specific transcriptional programs (Dogga et al., 2024; Jeninga et al., 2023). At the transcriptional level, sexual commitment is initiated by the master regulator AP2-G, while epigenetic factors such as HP1 and histone-modifying enzymes modulate chromatin accessibility and stabilize gametocyte- specific gene expression programs throughout development (Gomes et al., 2022; Parkyn Schneider et al., 2017).

Post-transcriptional regulation therefore adds an additional layer of control, allowing temporal and stage-specific modulation of gene expression. In the following sections, we focus on RNA methylation as a post-transcriptional epigenetic mechanism that is still relatively underexplored in malaria parasites, but for which emerging evidence points to an important role in modulating sexual differentiation, developmental timing and transmission competence.

Epigenetics refers to stable inherited phenotypic changes that regulate gene expression without altering DNA sequence, by controlling chromatin accessibility to transcriptional machinery (Waddington, 2012). It is a complex mechanism that includes histone modifications, chromatin structure changes, and non-coding RNA regulating chromatin accessibility (Nicoglou and Merlin, 2017).

Like the various chemical marks on histone tails, recent studies have also revealed several internal modifications in eukaryotic mRNA. These modifications include additional methylations of adenosine to form N1-methyladenosine (m1A) and N6,20-O-dimethyladenosine (m^6^Am), as well as methylation of cytosine to 5-methylcytosine and its oxidation product 5-hydroxymethylcytosine (hm^5^C). These observations suggest previously unrecognized regulatory roles of mRNA modification that may impact several cellular processes (Roundtree et al., 2017).

N^6^-methyladenosine (m^6^A) modification of mRNA constitutes a crucial layer of post-transcriptional regulation during the intra-erythrocytic developmental cycle, with the YTH domain protein PfYTH2 acting as a key “reader” that selectively binds m^6^A-modified transcripts, particularly near transcription termination regions (Govindaraju et al., 2020). Subsequent studies revealed that m^6^A levels and YTH readers are highly dynamic throughout blood-stage development and that PfYTH2 expression is even higher in gametocytes and salivary gland sporozoites (Baumgarten et al., 2019). PfYTH2 interacts with the translational machinery and functions as an m^6^A-dependent translational repressor (Sinha et al., 2014). A recent study indicates that the m^6^A–PfYTH2 axis is particularly important in male gametocytes, where it primes the transcriptome for rapid, temperature-dependent translational remodeling during mosquito transmission (Mussgnug et al., 2025). Together, these findings suggest that PfYTH2-mediated recognition of m^6^A-marked mRNAs, initially characterized in asexual blood stages, extends to sexual stages and contributes to the post-transcriptional control of gametocyte development and transmission competence.

Other studies have shown that m^6^A methylation is catalyzed by enzymes essential for the addition of a methyl group to the mRNA molecule, known as methyltransferases, or “writers,” which mediate the methylation process (Baumgarten et al., 2019; Arzumanian et al., 2022). These include: PfMT-A70, an analog of human methyltransferase-like 3 (METTL3), which acts as the primary catalytic enzyme in the reaction and; PfMT-A70.2, analogous to METTL14, which functions mainly as a regulator, playing a critical role in target RNA recognition and recruitment of the catalytic enzyme mentioned above. Another key component is the adaptor protein PfWTAP, an analog of WTAP (Wilms Tumor 1-Associating Protein), which serves as a scaffold to help properly position the writer complex on the correct mRNA sequence (Arzumanian et al., 2022; Liu et al., 2022).

Once these proteins mark a specific portion of RNA to be regulated, “reader” proteins interpret these modifications to determine whether the RNA fragment will be used, degraded, or stabilized (Catacalos et al., 2022). However, in *Plasmodium*, the identity of the proteins acting as readers remains unclear, with no definitive proteins described in the literature to date (Catacalos et al., 2022; Tang et al., 2025).

The stabilization of m^5^C transcripts likely plays a crucial role in the sexual stage development and transmission processes of *P. yoelii* and *P. falciparum*. Both species carry an ortholog of the NSUN2 gene (PyNSUN2; PfNSUN2) that functions as a methyltransferase, with a major role in m^5^C modifications of the transcriptome. *P. yoelii* gametocytogenesis is disrupted by the knockout (KO) of the Pynsun2 gene and restored by genetic complementation of Pynsun2 (Liu et al., 2022). NSUN2 is the main enzyme catalyzing m^5^C formation, while Aly/ REF export factor (ALYREF, an mRNA transport adaptor, also named THOC4) acts as a mRNA m^5^C-binding protein regulating mRNA export (Yang et al., 2017).

In *P. falciparum*, the DNMT2 homologue PfTRDMT1/PfDNMT2 functions as a tRNA methyltransferase that catalyzes the formation of 5-methylcytosine at position 38 (m^5^C38) in the anticodon loop of tRNA-Asp., as originally shown by Govindaraju et al. (2017). Building on this, Hammam et al. (2021) demonstrated that loss of PfDNMT2-mediated m^5^C38 methylation selectively downregulates GAC-codon-biased proteins, sensitizes parasites to metabolic and drug stress and, under nutrient stress, leads to degradation of m^5^C-deficient tRNA-Asp and a 6-fold increase in sexual commitment and gametocyte production. Together, these findings support the view that C38 m^5^C methylation in tRNA-Asp provides an additional epitranscriptomic layer of translational control over the Asp-biased proteome with direct consequences for parasite stress tolerance and the homeostasis of sexual differentiation.

## Epigenetic and RNA-binding machineries as therapeutic targets: current modulators and translational challenges

Epigenetic regulators that control sexual commitment and gametocyte maturation are attractive transmission-blocking targets because they sit upstream of the transcriptional and post-transcriptional cascades that define the fate of a subset of asexual parasites. In line with this, several classes of “epidrugs” originally developed in oncology have shown potent activity against *P. falciparum*, including sexual stages. Histone deacetylase (HDAC) inhibitors such as trichostatin A (TSA) and suberoylanilide hydroxamic acid (SAHA/Vorinostat) display nanomolar activity against early and late gametocytes and induce hyperacetylation of gametocyte histones, supporting an on-target effect on parasite HDACs and positioning this enzyme class as a bona fide gametocytocidal target (Trenholme et al., 2014). Building on these early proof-of-concept studies, more selective cyclic tetrapeptide HDAC inhibitors with improved *P. falciparum*–versus–host selectivity and fast-killing profiles have been developed, although issues such as reversible killing and residual host toxicity have so far limited their progression toward the clinic (Collins et al., 2021).

Likewise, screens of epigenetic libraries have identified inhibitors predicted to target histone methyltransferases and demethylases, some of which preferentially kill early or late gametocytes, underscoring that distinct epigenetic activities are differentially required across the gametocyte developmental window (Luo et al., 2023; Vanheer et al., 2020). These findings, together with systematic phenotypic profiling of epidrugs, support the view that epigenetic enzymes involved in sexual commitment, antigenic variation and stress responses constitute a rich reservoir of targets to develop multi-stage and transmission-blocking antimalarials (Reyser et al., 2024; Reyser et al., 2023).

Beyond global epigenetic modulation, deliberate rewiring of the AP2-G master regulator has been proposed as a conceptual “shock and kill” strategy. Because AP2-G activation represents a terminal commitment to sexual development, pharmacologically driving its expression, for example by inhibiting repressors such as HP1 or selected HDACs, could in principle force the bulk of the asexual parasite population into gametocytogenesis. As gametocytes do not replicate, this synchronized differentiation wave could debulk the symptomatic blood-stage infection, provided it is coupled with a potent gametocytocidal drug to remove these stages and avoid a transient surge in transmission potential (Reader et al., 2022).

Conversely, preventing AP2-G activation by stabilizing the heterochromatic silencing at its locus or inhibiting its activators (e.g., GDV1) would serve as a strict transmission-blocking intervention (‘lock’ strategy), essential for malaria elimination campaigns. While transcription factors like AP2-G have historically been considered ‘undruggable,’ emerging proteolysis-targeting chimeras and specific inhibitors of protein–protein interactions (e.g., disrupting the GDV1-HP1 interface) offer new possibilities for precise targeting of this commitment axis. In this context, a reporter-based phenotypic assay that quantitatively measures AP2-G–dependent sexual commitment could enable rapid screening of compounds that selectively modulate early gametocytogenesis without broadly affecting asexual growth.

Although no *P. falciparum*-specific small-molecule modulators of RNA-binding proteins or mRNA methylation have been reported to date, work in cancer has generated a first generation of tool compounds that conceptually extend to malaria. Inhibitors of the m^6^A “writer” METTL3 reduce global m^6^A levels and suppress the growth of acute myeloid leukemia, illustrating that the m^6^A machinery is chemically tractable *in vivo* (Yankova et al., 2021). Similarly, fragment-based and high-throughput approaches have yielded small-molecule inhibitors of YTH-domain m^6^A readers, including a recently described pan-YTH inhibitor that disrupts m^6^A-RNA binding in the low-micromolar range (Wang and Zhou, 2024). Given the conservation of key catalytic and aromatic cage residues between human and *Plasmodium* METTL3 orthologues and YTH-domain proteins, these scaffolds provide starting points to probe whether interference with PfMT-A70–PfWTAP complexes or PfYTH proteins can be harnessed to modulate sexual differentiation and gametocyte fitness. However, translating such compounds into parasite-selective antimalarials will require dedicated structure-guided optimization to exploit parasite-specific pockets while avoiding inhibition of host epitranscriptomic regulators.

Despite this promising preclinical landscape, several translational considerations currently limit the progression of epigenetic and RNA-binding pathways toward near-term clinical candidates against gametocytes. Similar to epigenetic regulators, many components of the RNA-binding and translational repression machinery (e.g., DDX6/DOZI, CITH, ALBA proteins, PUF proteins, and mRNA methylation readers and writers) share close functional or structural orthologs with host factors, raising potential concerns regarding parasite selectivity and host toxicity. In addition, these regulators often function within large and dynamic ribonucleoprotein assemblies, where context-dependent interactions and the absence of clearly defined catalytic sites may complicate conventional target-based drug discovery strategies. Furthermore, the deep-tissue sequestration of developing *P. falciparum* gametocytes, together with the current lack of standardized, high-throughput pharmacodynamic assays that directly measure gametocyte viability and transmission competence, poses additional challenges for prioritizing and validating transmission-blocking targets in this space (Josling and Llinás, 2015; Duraisingh and Horn, 2016).

Together, available evidence shows that epigenetic and post-transcriptional machineries are already being probed by first-generation small molecules with activity against asexual parasites, gametocytes and even quiescent, artemisinin-resistant forms, but converting these tool compounds into safe, parasite-selective transmission-blocking drugs will require integrated target-based and phenotypic screening in sexual stages, exploitation of parasite–host structural divergence, and rational combination regimens (Dai et al., 2024).

## Conclusion

**Figure 4:**
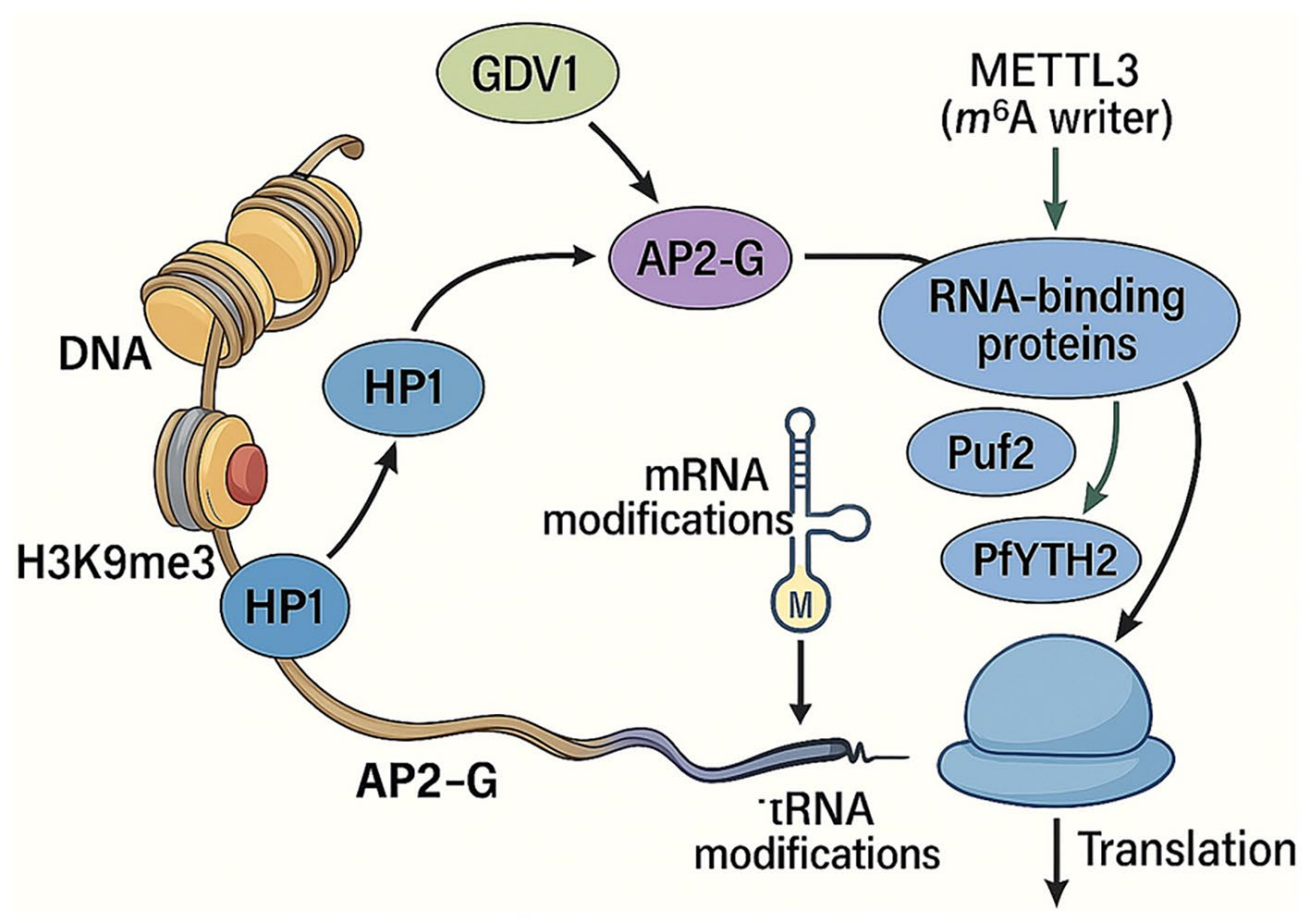
Molecular interplay among H3K9me3, HP1, GDV1, AP2-G, and RNA-binding proteins during commitment to gametocytogenesis. In asexual parasites, the *ap2-g* locus is embedded in heterochromatin marked by H3K9me3 and bound by HP1, maintaining transcriptional silencing. Upon induction of GDV1, HP1 is displaced from the chromatin, allowing transcriptional activation of AP2-G, the master regulator of sexual commitment. AP2-G mRNA and its downstream target transcripts are further controlled by RNA-binding proteins, including Puf2 and PfYTH2, which respond to mRNA and tRNA modifications such as METTL3-dependent m^6^A and PfDNMT2-linked marks, collectively reshaping the translational program that drives gametocyte development. Image generated by AI (ChatGPT).

As discussed in this review, gametocytogenesis is a complex process involving a wide range of genes and regulatory mechanisms (Figure 4). Understanding these processes is essential for identifying ways to regulate gametocytogenesis and its effective transmission to the mosquito vector, ultimately aiming to block malaria transmission. Although there has been a growing body of research on this topic, further investigations are still needed to identify new potential targets for drugs and vaccines, in order to translate these findings into practical applications for affected populations. The identification of gametocyte-specific genes and the discovery of new mechanisms of transcriptional and post-transcriptional regulation are crucial steps toward achieving this goal.

From a translational standpoint, the epigenetic and post-transcriptional pathways discussed here are already being interrogated by small-molecule modulators, including HDAC, DNMT and histone methyltransferase inhibitors, as well as emerging METTL3 and YTH-domain inhibitors developed in oncology. While these compounds provide invaluable tools and underscore the druggability of the underlying machineries, their limited parasite selectivity, pleiotropic effects and suboptimal pharmacokinetic profiles currently restrict direct repurposing for malaria and gametocytogenesis. Future efforts should therefore prioritize parasite-selective targeting of key regulators of sexual commitment and gametocyte maturation, the development of robust pharmacodynamic readouts for transmission-blocking activity, and rational combination strategies that integrate epigenetic or RNA-binding interventions with existing blood-stage drugs.

Looking ahead, emerging genetic and chemical tools provide concrete opportunities to advance epigenetic and RNA-binding machineries from conceptual regulators of gametocytogenesis to validated therapeutic targets. CRISPR-based strategies can be applied to test the druggability of key nodes that control sexual commitment and gametocyte maturation, as has been done with AP2-G, GDV1, chromatin modifiers and RNA-binding proteins, but there are still other challenges to drug development. By coupling these targeted perturbations with single-cell and chromatin-resolved approaches it will be possible to define additional regulators are not only essential for gametocyte development, but also create vulnerabilities that are amenable to pharmacological intervention.

In parallel, focused epigenetic probe sets and small-molecule libraries screened across asexual stages and well-characterized gametocyte populations can be used to identify novel modulators of histone acetylation and methylation, DNA and RNA methylation pathways, or mRNA–protein interactions that are critical for transmission. Hits from these screens will serve dual roles: as tool compounds to acutely interrogate target function in physiologically relevant models (including bone marrow–like niches and mosquito infection assays), and as starting points for medicinal chemistry programs aimed at parasite-selective, multi-stage and transmission-blocking agents. Integrating CRISPR-based target validation with stage-specific chemogenomic profiling and structure-guided optimization thus represents a rational framework to transform epigenetic and post-transcriptional regulators of gametocytogenesis into actionable therapeutic targets.
